# Cutting-Edge Therapies for Lung Cancer

**DOI:** 10.3390/cells13050436

**Published:** 2024-03-01

**Authors:** Anita Silas La’ah, Shih-Hwa Chiou

**Affiliations:** 1 Department of Medical Research, Taipei Veterans General Hospital, Taipei 112, Taiwan; anitalaah.ls07@nycu.edu.tw; 2 Taiwan International Graduate Program in Molecular Medicine, National Yang Ming Chiao Tung University and Academia Sinica, Taipei 115, Taiwan; 3 Institute of Pharmacology, School of Medicine, National Yang Ming Chiao Tung University, Taipei 112, Taiwan

**Keywords:** lung cancer, conventional treatment, innovative therapeutic modalities, combination therapy

## Abstract

Lung cancer remains a formidable global health challenge that necessitates inventive strategies to improve its therapeutic outcomes. The conventional treatments, including surgery, chemotherapy, and radiation, have demonstrated limitations in achieving sustained responses. Therefore, exploring novel approaches encompasses a range of interventions that show promise in enhancing the outcomes for patients with advanced or refractory cases of lung cancer. These groundbreaking interventions can potentially overcome cancer resistance and offer personalized solutions. Despite the rapid evolution of emerging lung cancer therapies, persistent challenges such as resistance, toxicity, and patient selection underscore the need for continued development. Consequently, the landscape of lung cancer therapy is transforming with the introduction of precision medicine, immunotherapy, and innovative therapeutic modalities. Additionally, a multifaceted approach involving combination therapies integrating targeted agents, immunotherapies, or traditional cytotoxic treatments addresses the heterogeneity of lung cancer while minimizing its adverse effects. This review provides a brief overview of the latest emerging therapies that are reshaping the landscape of lung cancer treatment. As these novel treatments progress through clinical trials are integrated into standard care, the potential for more effective, targeted, and personalized lung cancer therapies comes into focus, instilling renewed hope for patients facing challenging diagnoses.

## 1. Introduction

Lung cancer remains a significant cause of cancer-related deaths worldwide despite the progress made in cancer diagnosis and emerging treatment methods [1]. The 5-year overall survival rate for lung cancer patients is 19% across all stages of the disease. However, as the disease progresses from the early to advanced stages, there is a significant decline in the 5-year survival rate [2]. This is also observed in lung cancer patients with stage 1 tumors, where the 5-year recurrence-free survival only slightly exceeds 80% following curative surgical resection [1]. This implies that approximately 20% of individuals with lung cancer undergo disease recurrence within five years, underscoring the lack of a conclusive cure. Furthermore, a significant portion of lung cancer patients, constituting 57%, are diagnosed with metastasis, and their survival rate is as low as 5% [3].

Innovative approaches are continually shaping the landscape of lung cancer treatment, offering improved outcomes and novel options for patients. Advancements in this field encompass immunotherapy, targeted therapy, cryoablation, and the utilization of nanoparticle-based drug delivery systems, along with the evolving realm of gene therapy. However, ongoing research holds the promise of additional breakthroughs, contributing significantly to successful clinical outcomes that may revolutionize the care of lung cancer patients [4,5,6]. Combinatorial therapeutic approaches represent a significant spectrum of innovative strategies. The ESMO Congress 2023 notably highlights the efficacy of combining targeted drugs and immunotherapy, especially for lung cancer patients with EGFR mutations and rare tumor alterations. These advancements are poised to play a substantial role in revolutionizing the landscape of lung cancer treatment. This review presents a concise summary of such innovative therapeutic approaches.

## 2. Targeted Therapies

Molecular alterations such as EGFR mutations, ALK rearrangements, ROS1 rearrangements, RET rearrangements, NTRK fusions, MET mutations, KRAS mutations, BRAF V600E mutations, and HER2 mutations are the contributing factors that underlie the aggressiveness of lung cancer. Therefore, targeted therapies are designed to overcome the consequences of these mutations and primarily achieve great success in the treatment and prognosis of this formidable disease, resulting in an improved survival rate [7]. Despite the emergence of such therapies, significant challenges remain, as oncogenic driver mutations lack specific targeted agents, and resistance is a recurring issue.

### 2.1. Epidermal Growth Factor Receptor (EGFR) Inhibitors

Epidermal growth factor receptor (EGFR)-activating mutations are prevalent in non-small cell lung carcinoma (NSCLC), which is the predominant type of lung cancer. Around 4–10% of NSCLC patients with EGFR mutations exhibit EGFR exon 20 insertion (ex20ins) mutations, whereas 46% have EGFR exon 19 deletion (ex19del) mutations and 38% harbor the EGFR L858R point mutation [8]. The discovery of tyrosine kinase inhibitors (TKIs) designed to target EGFR mutations in lung cancer patients marked the inception of the precision medicine era in lung cancer. EGFR TKIs have been designed to target these mutations effectively by inhibiting the activation of the tyrosine kinase domain and disrupting various EGFR-dependent/independent downstream signaling pathways in the lungs [9]. Currently, there are three generations of clinically available EGFR TKIs, namely the first generation of reversible inhibitors (gefitinib, erlotinib, and icotinib), the second generation of irreversible inhibitors (afatinib, dacomitinib), and the third generation of irreversible inhibitors (osimertinib, almonertinib, and lazertinib) [9]; some of these inhibitors are listed in Table 1. Although the first- and second-generation TKIs effectively overcome the effects of EGFR mutations, resistance inevitably develops, primarily due to the emergence of the T790M mutation [10,11]. To overcome this major challenge, osimertinib, a third-generation TKI, has been recommended as the first-line treatment for advanced NSCLC with activated EGFR mutations, irrespective of T790M status [12,13,14]. A clinical trial (NCT02296125) evaluating osimertinib demonstrated notable efficacy with reduced adverse effects in NSCLC patients when compared to the conventional EGFR TKIs administered as the primary treatment [15]. Nevertheless, lung cancer patients treated with osimertinib eventually develop acquired resistance, which is characterized by different mechanisms, either according to EGFR-dependent or EGFR-independent pathways [11,16,17,18]. To address this acquired resistance, developing BBT-176 as a fourth-generation EGFR TKI is considered effective against the C797S mutation, which confers resistance to third-generation TKIs [19]. Combination strategies are undergoing preclinical and clinical investigations to address EGFR TKI resistance. Among the clinical trials are I-PASS (the combination of gefitinib vs. carboplatin with paclitaxel to target exon 18–21 mutations) [20], WJTOG3405 (the combination of gefitinib vs. cisplatin with docetaxel to target exon 19 and 21 mutations) [21], and FLAURA (the combination of osimertinib with gefitinib or erlotinib to target exon 19 and 21 mutations) [15].

Monoclonal antibodies offer an alternative strategy for inhibiting EGFR activation and signaling. These antibodies not only form complexes with the receptor, which are internalized and eliminated, but can also entirely block ligands from attaching to the extracellular domain. The available monoclonal antibodies targeting EGFR include cetuximab, necitumumab, panitumumab, and matuzumab. In two phase III trials, FLEX and BMS099, a combination of cetuximab and platinum doublet chemotherapy was employed to treat advanced NSCLC [22,23]. Additionally, amivantamab, a bispecific antibody targeting both EGFR and MET, was used to treat NSCLC specifically associated with EGFR exon 20 insertions [24]. In a preclinical study, the combination of amivantamab and lazertinib, which targets both the EGFR extracellular and catalytic domains, demonstrated synergistic tumor growth inhibition [25].

EGFR TKIs significantly enhance the objective response rate, progression-free survival, and quality of life when compared to conventional chemotherapeutic approaches, all while presenting minimal toxicity [26,27]. The adoption of EGFR TKIs marks a significant leap forward in the treatment of NSCLC, ushering in an era of targeted therapy and precision medication.

**Table 1:** Representative drugs that target EGFR mutations in lung cancer.

Representative Drugs	Target Mutation Site	Approved Indication	OS (Months)	Refs.
Gefitinib	EGFR Ex19del, L858R	Advanced or metastatic NSCLC	30.5–38.8	[21,28]
Erlotinib	EGFR Ex19del, L858R	Advanced NSCLC	19.3	[29,30,31]
Icotinib	EGFR Ex19del, L858R	Advanced NSCLC	30.5	[32,33]
Afatinib	EGFR Ex19del, L858R	Locally advanced or metastatic NSCLC	19.6–27.6 * 30.7–33.3 *	[34,35,36]
Dacomitinib	EGFR Ex19del, L858R	Advanced or metastatic NSCLC	34.1	[37,38]
Osimertinib	EGFR Ex19del, L858R, T790M	Advanced or metastatic NSCLC	38.6	[15,39]
Aumolertinib	EGFR Ex19del, L858R, T790M	Advanced NSCLC	NR	[40]
Mobocertinib	EGFR exon 20 insertion	Advanced or metastatic NSCLC	24.0	[41]
Cetuximab	EGFR	Advanced NSCLC	10.9	[42]
Amivantamab	EGFR exon 20 insertion	Advanced NSCLC	11.4	[43]

* The table summarizes some drugs designed to target EGFR mutation with ther overall survival (OS) in lung cancer.

### 2.2. Kirsten Rat Sarcoma Viral Oncogene Homologue (KRAS) Inhibitors

Kirsten rat sarcoma viral oncogene homologue (KRAS) is a well-known oncogene encoding the Ras family of small GTPases, controlling crucial proliferation and survival pathways. Among three members of the Ras family, KRAS is the most frequently mutated in cancers (85%), followed by NRAS (11%) and HRAS (4%). The most frequent KRAS-activating mutations occur at the amino acid positions G12, G13, and Q61 [44]. The Ras oncogenes play a crucial role in oncogenesis and have been naturally considered potent targets for cancer therapy. However, several efforts to target Ras proteins have faced considerable challenges due to molecular features such as a highly dynamic structure and high intrinsic flexibility precluding stable binding of the inhibitors, thus deeming them “undruggable” [45]. However, new technologies and insights into the KRAS signaling pathways have renewed efforts to develop therapies for KRAS-driven cancers. These include direct KRAS targeting or indirect targeting by blocking the upstream factors activating KRAS [46].

A direct approach to targeting KRAS in lung cancer involves using sotorasib (AMG510) and adagrasib (MRTX849). Sotorasib is a covalent inhibitor designed for KRAS G12C and marked a milestone as the first KRAS inhibitor to receive US Food and Drug Administration (FDA) approval on 28 May 2021 [47]. This drug covalently binds to the mutant cysteine 12 in the switch II region, prompting KRAS to stay inactive in its GDP-bound form. Consequently, it inhibits KRAS signaling and suppresses the MAPK pathway. In a phase II clinical trial encompassing 126 patients with advanced NSCLC, sotorasib demonstrated a 37.1% response rate, a progression-free survival of 6.8 months, and a median overall survival of 12.5 months [48]. Adagrasib is another FDA-approved small molecule directly targeting KRAS G12C by covalently binding to the mutant cysteine 12, effectively inhibiting KRAS-dependent signaling, such as the MAPK pathway [44]. Clinical trials are actively exploring the potential of other drugs that covalently inhibit KRAS G12C, such as divarasib (GDC-6036) from Genentech, which is currently undergoing a phase 1 clinical trial (NCT04449874) as a monotherapy and in combination with other anticancer therapies. This research is being conducted on patients with advanced/metastatic solid tumors carrying a KRAS G12C mutation [45].

Another chemotherapeutic approach is to target KRAS indirectly by inhibiting its upstream regulators. Currently, a phase I clinical trial (NCT04111458) is evaluating the efficacy of BI1701963, an inhibitor of SOS1, which serves as a guanine nucleotide exchange factor, turning KRAS into its GTP-bound active form. This study investigates the effectiveness of BI1701963 both as a monotherapy and in combination with the MEK inhibitor trametinib [49]. Additionally, Novartis Pharmaceuticals is conducting a phase I/II clinical trial (NCT04699188) to assess the effectiveness of TNO155, an inhibitor of tyrosine phosphatase SHP2, which serves as another upstream factor responsible for KRAS activation. It is being tested as both monotherapy and in combination with spartalizumab anti-PD1 antibody immunotherapy in patients with advanced or metastatic solid tumors featuring the KRAS G12C mutation [45]. Jacobio Pharma is also actively investigating the efficacy of JAB-21822, an inhibitor of KRAS G12C, in various clinical trials, either as a monotherapy or in combination with the JAB-3312 SHP2 inhibitor or cetuximab anti-EGFR immunotherapy [50].

To summarize, direct targeting of KRAS has been challenging due to its high affinity for GTP at the picomolar level, the absence of appropriate pockets for high-affinity small-molecule binding, and its elevated mutation rate. However, the covalent modification of the mutated cysteine 12 in KRAS G12C has proven to be a viable option. Despite this, cancer cells still develop resistance to these inhibitors, prompting the exploration of combination therapies and new approaches to treating KRAS-driven cancers.

### 2.3. Anaplastic Lymphoma Kinase (ALK) Inhibitors

The anaplastic lymphoma kinase (ALK) receptor tyrosine kinase plays a pivotal role in cellular development, and alterations in the ALK gene may occur in cancers such as anaplastic large cell lymphoma, neuroblastoma, and NSCLC. When the ALK gene is activated in cancer, it can lead to cell development and rapid growth. This activation of ALK signaling in the tumor cells is brought about by mechanisms such as gene fusions, chromosomal translocations, gene amplification or deregulation, and activating point mutations [51,52]. In treating NSCLC patients with ALK alterations, targeted inhibitors such as crizotinib, ceritinib, alectinib, brigatinib, and lorlatinib offer significant benefits [53]. Crizotinib is a potent small-molecule drug that effectively targets the tyrosine kinases ALK and c-MET [54]. Phase I/II clinical studies have demonstrated that crizotinib enhances progression-free survival in combination with bevacizumab, an angiogenesis-inhibiting antibody [55]. On the other hand, ceritinib, a second-generation ALK inhibitor, has been utilized to treat advanced or metastatic ALK-positive NSCLC, even in patients resistant to crizotinib [56,57]. Despite the initial effectiveness of the existing ALK inhibitors, resistance inevitably develops. SAF-189s is a novel ALK inhibitor that has shown promise in overcoming the majority of the known resistance mutations associated with ALK in preclinical studies and is currently undergoing a phase I/II study in China [58]. APG-2449 is a triple kinase inhibitor of ALK, ROS1, and FAK that has shown anti-tumor activity in a mouse model of ALK/ROS1-positive NSCLC. Currently, it is undergoing evaluation in a phase I dose escalation and expansion trial (NCT03917043), which enrolled 84 patients diagnosed with ALK/ROS1-positive NSCLC [59].

### 2.4. ROS Proto-Oncogene 1, Receptor Tyrosine Kinase (ROS1) Inhibitors

ROS proto-oncogene 1, receptor tyrosine kinase (ROS1) is a paralog of ALK belonging to the insulin receptor family that functions as a growth or differentiation factor receptor. The incidence of ROS1 rearrangements is observed in 1% to 2% of NSCLC cases. Although some ROS1 inhibitors, specifically crizotinib (first generation), entrectinib (second generation) and lorlatinib (third generation), have received FDA approval for treating ROS1-positive NSCLC, the majority of patients still encounter challenges with treatment resistance and disease progression [27,60]. Clinical investigations have evaluated the effectiveness of additional inhibitors in patients with ROS1-positive NSCLC, including repotrectinib [61] and taletrectinib [62]. Both of these inhibitors received FDA approval in 2022 [7].

### 2.5. BRAF V600E Mutation Inhibitors

BRAF mutations are rare mutations in NSCLC, with a higher prevalence observed in never-smokers, women, and aggressive histological types, particularly the micropapillary subtype [63]. Cancer cells harboring the V600E BRAF mutation rely predominantly on the activity of this oncogene for their growth and survival [64]. Some BRAF V600E mutation inhibitors are vemurafenib, dabrafenib, and sorafenib. Clinical investigation has demonstrated that vemurafenib, a potent inhibitor of the BRAF V600E mutation, exhibits an antitumor effect in NSCLC [65]. Furthermore, dabrafenib demonstrated enhanced efficacy in treating advanced NSCLC characterized by the BRAF V600E mutation [66]. Combination therapy strategies have enhanced the treatment efficacy in lung cancer patients harboring the BRAF V600E mutation. For instance, dabrafenib plus trametinib in treating metastatic NSCLC with BRAF V600E has shown significant and lasting clinical benefits, alongside a manageable safety profile [67]. Moreover, the FDA recently approved the combination of encorafenib with binimetinib for metastatic NSCLC harboring the BRAF V600E mutation, with clinical trials demonstrating its notable effectiveness in NSCLC treatment [68].

### 2.6. Human Epidermal Growth Factor Receptor (HER2 or ERBB2) Mutation Inhibitors

In lung cancer, approximately 90% of HER2 mutations consist of in-frame non-frameshift insertions located in exon 20 of the tyrosine kinase domain (ex20ins) [69]. The discovery of HER2 provides hope for lung cancer patients with HER2 abnormalities [70]. Monoclonal antibodies play a vital role in anti-HER2 therapy, with trastuzumab deruxtecan (T-DXd, DS-8201) standing out as a notable example that has shown encouraging antitumor effects in HER2-mutant lung cancer patients. Furthermore, pan-HER TKIs like afatinib, neratinib, and dacomitinib have been used to treat patients with HER2-mutant NSCLC [71]. Selective HER2 tyrosine kinase inhibitors (TKIs) such as pyrotinib, poziotinib, mobocertinib, tarloxotinib, afatinib, neratinib, and dacomitinib have been employed in lung cancer treatment [72]. For instance, clinical studies have indicated that pyrotinib can enhance the overall survival of NSCLC patients with HER2 mutations, regardless of metastatic status [73]. Moreover, a multicenter and multi-cohort phase II study (ZENITH20) was conducted to evaluate the efficacy of poziotinib as a monotherapy in NSCLC. The findings demonstrated moderate effectiveness in HER2-mutant NSCLC [74].

## 3. Immunotherapy

Immunotherapy has markedly reshaped cancer treatment, owing to its well-tolerated safety profile, capacity to induce enduring therapeutic responses through the generation of immunological memory, and effectiveness across a broad spectrum of patients [75]. Various emerging methods in lung cancer immunotherapy include tumor-specific vaccination strategies, immune checkpoint inhibitors, adoptive cell therapy, etc. [76]. Several clinical trials have been conducted to assess the efficacy of immunotherapy in lung cancer, specifically in patients facing challenges that involve the absence of a targetable driver mutation [5]. Cancer immunotherapies are carried out mainly to impede tumor growth and enhance the survival outcomes of patients by boosting the host’s anti-tumor immunity and modifying the suppressive tumor microenvironment [77].

### 3.1. Adoptive Cell Transfer

Adoptive cell transfer (ACT) for lung cancer involves extracting T cells from the patient’s bloodstream [78]. An example of adoptive cell transfer is CAR-T cell therapy, which involves the genetic modification of T lymphocytes from lung cancer patients to make them express chimeric antigen receptors (CARs) [79]. Such CAR-T cells are introduced back into the body, and the CARs recognize the antigens expressed by cancer cells, which trigger their destruction [80]. Currently, the research on CAR-T cell therapy for lung cancer is in its initial exploration phase. Despite numerous clinical trials, there are several challenges to address, such as on-target/off-tumor toxicity, tumor antigen variability, the immunosuppressive tumor microenvironment, neurological toxicity, and cytokine release syndrome. Addressing these challenges represents the forefront of the research in CAR-T cell therapy for lung cancer [81]. Furthermore, tumor-infiltrating lymphocytes (TILs) represent another type of adoptive cell transfer application that entails isolating TILs from the tumor site through biopsy or surgery. Subsequently, these isolated TILs are stimulated with interleukin-2 (IL-2) and reintroduced into the patient through infusion, aiming to target and attack the cancer cells [82].

### 3.2. Immune Checkpoint Inhibitors

Checkpoint proteins such as programmed cell death protein 1 (PD-1), programmed death-ligand 1 (PD-L1), and cytotoxic T-lymphocyte associated protein 4 (CTLA-4) constitute a restraining mechanism of the immunity system that prevents it from autoimmune reactions but, at the same time, is related to immune escape by cancer cells. Dysregulation in these pathways is associated with immune escape and increased cancer progression [83]. Immune checkpoint inhibitors have demonstrated impressive clinical effectiveness and safety in the treatment of lung cancer, leading to their incorporation across all stages of managing NSCLC using both adjunctive (e.g., atezolizumab) and pre-adjunctive (such as nivolumab) therapies [84]. For instance, pembrolizumab (Keytruda) and nivolumab (Opdivo), antibodies designed against PD-1 and CTLA-4, demonstrated effectiveness in lung cancer treatment [85]. Some of the immune checkpoint inhibitors applied to lung cancer therapy are listed in Table 2.

### 3.3. Cancer Vaccines

DNA and mRNA vaccines for cancer have become a promising strategy for both prevention and treatment. This method includes the introduction of DNA or RNA sequences that encode tumor-associated antigens (TAAs) or neoantigens, resulting in specific targeting of cancer cells [93]. Despite being in the early stages of development and clinical testing, cancer therapeutic vaccines, particularly those designed for lung cancer, show potential in treating patients resistant to the standard-of-care treatment [94]. Clinical trials on cancer therapeutic vaccines are faced with challenges due to the cost of tissue processing, the identification of proteins/peptides, and vaccine formulation in NSCLC [95]. Nevertheless, in a phase III clinical trial (NCT00409188), the utilization of the cancer vaccine Stimuvax^®^ (tecemotide; L-BLP25 or BLP25 Liposome Vaccine) following chemoradiotherapy showed no significant impact on patients compared to a placebo in stage III NSCLC [96].

### 3.4. Oncolytic Viruses (OVs)

In lung cancer treatment, oncolytic viruses (OVs) operate by selectively identifying, infecting, and eliminating cancer cells while minimizing the harm to healthy cells [97]. The main mechanism of OVs involves inducing specific antitumor immune responses and selective cell death, resulting in tumor cell lysis and a reduction in tumor progression [98]. Currently, clinical trials are ongoing to evaluate the effectiveness of the following OVs in treating lung cancer: RT-10 (NCT05205421), ADV/HSV-tk (NCT03004183), MEM-288 (NCT05076760), and YSCH-01 (NCT05180851) [99]. A clinical investigation demonstrated that Oncorine (previously known as H101), a modified human adenovirus type 5, caused stable disease with partial necrosis of the lung tissue when combined with nivolumab and anlotinib in a recurrent NSCLC patient who had developed resistance to nivolumab [100]. Additionally, the administration of Reolysin with paclitaxel and carboplatin resulted in a 30% improvement in the survival rate of patients with metastatic/recurrent NSCLC harboring KRAS mutations or EGFR mutations/amplifications [101]. Hence, the utilization of OVs in lung cancer treatment holds the potential to improve the overall patient outcomes.

### 3.5. Targeting Immune Checkpoint Receptors (ICRs)

Targeting immune checkpoint receptors (ICRs) such as lymphocyte activation gene-3 (LAG-3), T cell immunoglobulin and mucin-domain-containing-3 (TIM-3), and T cell immunoreceptor with immunoglobulin and tyrosine-based inhibitory motif (ITIM) domain (TIGIT) represent a promising immunotherapy for lung cancer treatment. Clinical trials are ongoing to investigate the efficacy of targeting LAG3, TIM-3, and TIGIT in lung cancer. For instance, a clinical trial (NCT02964013; MK-7684-001) evaluating the anti-TIGIT (T cell immunoglobulin and ITIM domain) antibody vibostolimab in combination with pembrolizumab revealed significant anti-tumor activity compared to vibostolimab monotherapy in advanced NSCLC [102]. Furthermore, in a phase I/II trial (NCT02608268), MGB453 (an anti-TIM3 agent) was assessed in combination with PDR001 (an anti-PD-1 therapy) for advanced solid cancers, including NSCLC and melanoma. The trial demonstrated a favorable safety profile, with anti-tumor activity [103]. Despite the absence of FDA-approved therapies targeting TIM-3, the advancement of novel TIM-3 inhibitors is rapidly evolving in lung cancer. Numerous TIM-3-specific antibodies, such as cobolimab, sabatolimab (MBG453), BMS-986258, AZD7789, INCAGN02390, etc., are under investigation [104].

## 4. Radiation Therapy

Radiotherapy is a conventional method employed in the treatment of lung cancer; however, a notable challenge lies in the deposition of doses into healthy tissues before reaching the intended targets, and this is likely to cause severe complications in patients with recurrent cancer. This necessitates the development of more targeted approaches, allowing the specific destruction of cancer while sparing healthy tissues. Some promising approaches in radiation therapy are listed below.

### 4.1. Intensity-Modulated Radiation Therapy (IMRT) and Volumetric-Modulated Arc Therapy (VMAT)

Intensity-modulated radiotherapy (IMRT) and volumetric-modulated arc therapy (VMAT) represent advanced radiotherapeutic modalities that have improved dosimetric outcomes in lung cancer treatment [105]. This technique directs high-dose radiation to targeted disease sites by effectively minimizing the exposure to the neighboring organs at risk [106]. Clinical studies have been carried to investigate the efficacy of either IMRT or VMAT and IMRT/VMAT in lung cancer. For instance, a hybrid technique incorporating two partial arcs of VMAT along with a five-field IMRT approach was developed for 15 NSCLC patients. This hybrid IMRT/VMAT method notably enhanced both the target dose conformity and homogeneity, demonstrating superior efficiency compared to standalone IMRT and VMAT techniques [107]. Moreover, clinical investigations have shown that incorporating hybrid VMAT into advanced lung cancer treatment enables the safe administration of 60–66 Gy while reducing the volume of low-dose radiation exposure to the lungs [108].

### 4.2. Boron Neutron Capture Therapy (BNCT)

Boron neutron capture therapy (BNCT) is a radiation therapy method that selectively targets and eliminates cancer cells while sparing normal cells [109]. This method is based on the preferential accumulation of compounds containing the boron isotope ^10^B in cancer cells. Upon exposure to a beam of low-energy neutrons, ^10^B is converted into unstable ^11^B, which decays into α particles (4He) and 7Li recoil particles. The high-energy particles generated as a result of the boron–neutron interaction exhibit a limited impact range, primarily affecting the cells in which boron is concentrated. This leads to localized damage to the cancer cells, sparing the surrounding healthy tissues [109].

BNCT is a pivotal treatment option, offering selectivity and reduced toxicity for lung cancer and metastatic lung disease [110]. Previous studies have shown that BNCT exhibited minimal toxicity and effectively suppressed lung metastases within a short treatment period in a BDIX rat model with lung metastases of colon carcinoma [111,112]. BNCT mediated by 10B-carrier L-para-boronophenylalanine-10B (BPA) treatment was also monitored in normal lungs of Fischer 344 rats by assessing the established relative biological effectiveness (RBE) and compound biological effectiveness (CBE) factors [113]. This method was employed in patients with recurrent lung cancer who had previously undergone chest wall irradiation with two fractions of BNCT. The tumor exhibited regression within seven months, with minimal or delayed adverse effects [114]. Additionally, two patients diagnosed with malignant pleural mesothelioma and malignant short spindle cell tumors underwent BNCT. Subsequently, both patients demonstrated either tumor regression or stability in tumor size for 3–6 months, without any reported adverse events [115].

**Figure 1:**
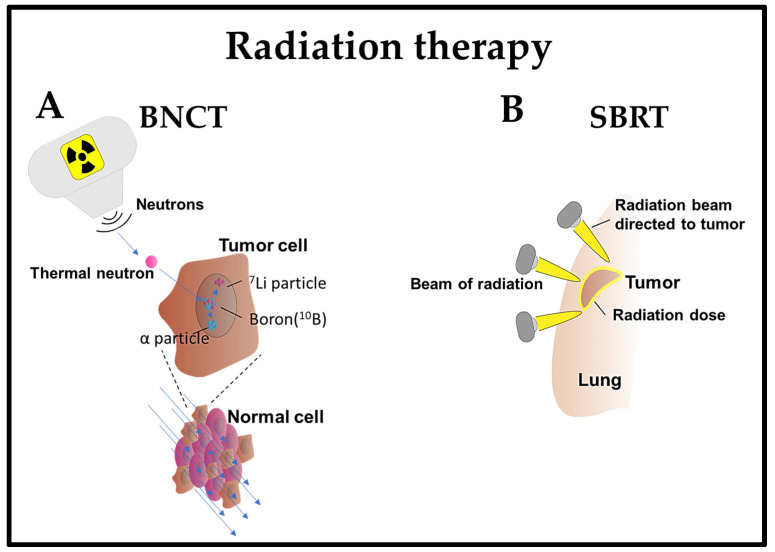
Emerging radiotherapy strategies. (**A**) BNCT: After the administration of a non-radioactive compound containing the inert isotope ^10^B, which is specifically homed in cancer cells, the patient is exposed to a low-energy neutron beam. This beam initiates the fission of the ^10^B isotope within the tumor cells, leading to the emission of a high-energy α-particle. This particle selectively kills cancer cells containing the ^10^B isotope compound; (**B**) SBRT: A four-dimensional CT scan is employed to observe the movement of lung cancer during inhalation and exhalation. High-dose radiation beams from various angles are then precisely directed at the tumor.

The integration of BNCT with additional therapeutic modalities has been explored in mouse model studies. Specifically, the combination of BPA-mediated BNCT with mild temperature hyperthermia and the hypoxic cytotoxin tirapazamine (TPZ), targeting the quiescent tumor cell population, significantly reduced lung metastases [116] (Figure 1).

### 4.3. Stereotactic Body Radiation Therapy (SBRT)

Stereotactic body radiation therapy (SBRT) is an efficient and potentially effective option for treating inoperable early-stage NSCLC patients [117] or any stage of lung cancer [118]. It is a non-invasive treatment that delivers high doses of radiation with precision in a few treatments, achieving superior local control and survival rates compared to conventional radiation therapy [119]. SBRT utilizes sophisticated imaging and localization techniques to enhance the precision of radiotherapy targeting. This optimization enables the administration of hypofractionated and ablative doses of radiation [120]. The effectiveness of SBRT lies in its ability to deliver therapeutic radiation doses with a relatively high probability of tumor control while minimizing the exposure of normal tissue to these doses [121]. For instance, patients diagnosed with lung cancer, regardless of whether they underwent prior lung resection or not, receiving SBRT demonstrated high local control and relatively low toxicity [122].

SBRT has been employed in 19 prospective clinical trials for primary early-stage NSCLC, encompassing 1434 patients with central and peripheral early-stage NSCLC. The findings revealed that SBRT demonstrated excellent local and regional control, with the survival rates ranging from 43% to 95% at three years. Nevertheless, up to 33% of patients experienced distant failure following SBRT. The treatment was generally well tolerated, with 10–30% of patients encountering grade 3–4 toxicities and a limited number of treatment-related deaths. No discernible differences in outcomes were noted between conventional fractionated radiation therapy and SBRT or between central and peripheral lung tumors, as well as between inoperable and operable patients [123].

SBRT is gaining prominence in intricate cases, including patients with tumors situated close to vital organs, those with a history of previous radiation exposure, individuals with interstitial lung disease (ILD), or patients with metastatic disease. In instances of ultracentral tumors (those close to the trachea or proximal bronchial tree), SBRT poses an increased risk of severe toxicity, encompassing pulmonary hemorrhage or airway necrosis [119]. SBRT remains a viable treatment choice for medically inoperable and operable patients diagnosed with early-stage NSCLC, providing excellent local and regional control, accompanied by lower toxicity rates [124] (Figure 1).

## 5. Cryoablation

Cryoablation is a therapeutic approach that destroys tumors using extreme cold [125]. This process involves connecting cryoprobes to pressurized argon, which rapidly cools the probe upon its expansion to temperatures as low as −160 °C. Consequently, this results in the formation of an ice ball at the tip of the cryoprobe. The freezing and thawing process disrupts the cell membrane and initiates microvascular injury, subsequently inducing hypotonic stress and leading to cell necrosis [126]. In lung tumors, cryoablation is typically conducted with the guidance of CT scans, accompanied by sedation and local anesthesia [125]. The procedure can be performed via endobronchial, direct intrathoracic, or percutaneous routes, depending on the location and size of the tumor [127]. Some adverse effects faced by lung cancer patients include mild complications such as bleeding, pneumothorax, and pulmonary infection [125].

Typically, patients with lung metastases frequently struggle to attain curative results despite undergoing chemotherapy, radiotherapy, or surgery [126]. However, studies indicate that cryoablation can potentially treat lung metastasis effectively [128]. A promising strategy involves combining cryoablation with immunotherapy; however, cryosurgery alone cannot elicit a robust immunotherapeutic response to cancer [126]. The administration methods for combining cryoablation with immunotherapy include percutaneous and bronchoscopic approaches [127]. Clinical trials were carried out to explore the effectiveness of combining cryosurgery with allogeneic NK cell immunotherapy for treating NSCLC. The research revealed improved immune function in patients, leading to significantly higher response rates and disease control rates compared to the cryoablation-only group [129]. Furthermore, in advanced NSCLC patients, the therapeutic impact of cryoablation was elevated when combined with gefitinib [130]. Comparative studies on NSCLC indicated that both cryoablation and microwave ablation were safe and effective for small tumors. Nevertheless, microwave ablation demonstrated a superior effect when dealing with larger tumors [131]. These recent extended follow-up studies suggest that cryoablation is emerging as a notable choice for diverse cancers, providing the prospect of prolonged survival (Figure 3).

## 6. Photodynamic Therapy (PDT)

Photodynamic therapy (PDT) is a non-invasive lung cancer treatment that utilizes photosensitive compounds and light activation to selectively destroy cancer cells [63]. PDT has demonstrated efficacy in enhancing the survival rate of patients with incurable malignancies by using three fundamental factors: photosensitizer drugs, light, and oxygen [132]. Photosensitizers exert their photodynamic activity through photo-oxidative mechanisms, triggering diverse biochemical and morphological reactions that lead to cytotoxic effects in tumors [133]. For instance, chlorin e6 (Ce6) is a frequently used photosensitizer that causes DNA damage and elicits a DNA damage response (DDR) in lung cancer cells. When such PDT treatment is combined with the inhibition of ATM serine/threonine kinase responsible for DDR, lung cancer cells are effectively destroyed [134]. However, the effectiveness of PDT is primarily constrained by the lipophilic nature of the photosensitizers and their limited tissue infiltration [135]. The applicability of PDT tends to be limited to superficially located tumors which can be easily accessed using a bronchoscope with a light source [136]. For instance, it can be used as a complementary measure to treat advanced-stage lung cancers in which the tumors have spread to bronchoscope-accessible areas. In such cases, it is typically employed alongside chemotherapy and radiation, rather than as a replacement [137].

The integration of nanotechnology into PDT has the potential to surmount its limitations by facilitating drug delivery and release [138]. This can result in more efficient and precisely targeted treatment approach to lung cancer [139]. For instance, Ce6-conjugated methoxy-poly (ethylene glycol)-b-poly (D, L-lactide) (mPEG-PLA-Ce6) amphiphilic polymer nanoparticles were utilized as Ce6 carriers in PDT and elicited enhanced phototoxicity and efficient internalization in both the monolayers and 3D spheroids of human lung adenocarcinoma cells [140]. In another study, mesenchymal stem cells (MSCs) characterized by high tumor tropism were loaded with MnO_2_@Ce6 nanoparticles to precisely deliver photosensitizers to lung cancer sites and target them for PDT in a mouse model [141].

Several PDT clinical trials have been undertaken; the most recent significant study was centered on the combination of Laserphyrin^®^-based PDT and chemotherapy for advanced NSCLC cases in which curative surgical interventions were not feasible. The aim was to address bronchial stenosis and obstruction in the central and peripheral (lobar or segmental bronchi) lung areas, and PDT resulted in improved symptoms and quality of life [142]. Additionally, second-generation Radachlorin^®^-based PDT was employed for advanced NSCLC, resulting in a one-year post-treatment survival rate of 70%, with improved treatment effectiveness and safety [143]. A phase 1 clinical trial of PDT targeting carcinomas in situ and microinvasive carcinomas in the central airways was carried out with the use of 2-[1-hexyloxyethyl]-2-devinyl pyropheophorbide-a (HPPH) as the photosensitizer. This approach demonstrated safety and efficacy in treating NSCLC by attaining a complete response (CR) rate of 72.7% at six months [144]. Furthermore, integrating high dose rates of BT and PDT for endobronchial tumors at different stages (I–IV) has proven to be well tolerated. This combined approach achieved prolonged local control and maintained an acceptable level of morbidity [145].

**Figure 2:**
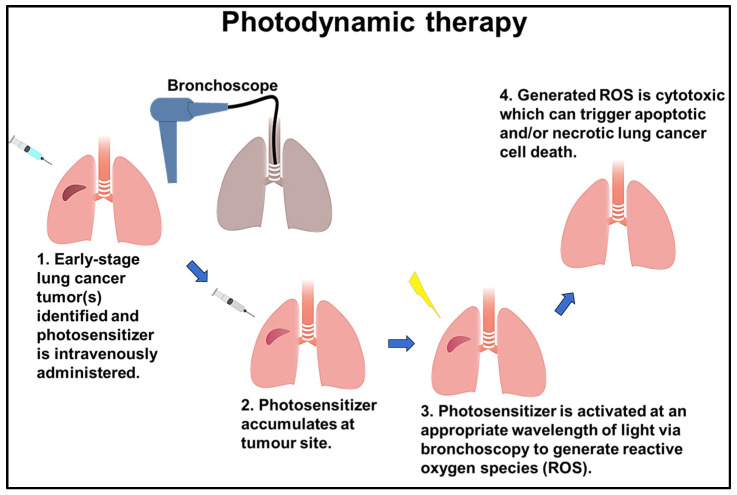
Simplified photodynamic therapy steps: (1) the patient receives a photosensitizer (PS) drug through injection near the tumor; (2) the PS drug gathers at the tumor site, either actively or passively; (3) after absorption by the tumor, laser light at the right wavelength (630 nm–780 nm) is used to activate the PS; (4) activation results in targeted destruction of the tumor cells.

A novel PDT technique, intelligent targeted antibody phototherapy (iTAP), has recently been proposed. This method is based on the combination of cetuximab immunotoxin treatment with mono-L-aspartyl Pe6 (NPe6)-based PDT and was shown to be a minimally invasive yet highly effective treatment compared to conventional PDT [146] (Figure 2).

## 7. Hyperthermia Therapy

Hyperthermia therapy (HT), or thermal therapy, is a cancer treatment involving artificial elevation of the body tissue temperature. This is accomplished by administering heat from external sources such as microwaves, radio waves, lasers, ultrasound, etc., to locally elevate the temperature to 42–45 °C. This process aims to eliminate cancer cells or inhibit their growth without causing harm to normal tissues [147]. HT induces direct cytotoxic effects in lung cancer cells [148], as well as enhancing tumor perfusion, thus increasing the drug delivery capability [149]. At temperatures above 42 °C, tumor blood vessels can collapse, trapping the applied heat and leading to necrosis or apoptosis [150]. On the contrary, temperatures below 42 °C influence the tumor microenvironment and increase blood flow and vascular permeability, thus potentially improving the supply of oxygen and nutrients to the tumor cells. This makes precise temperature control a strict consideration for achieving the desirable effect of HT. For instance, an increase in perfusion acts as a coolant that dissipates the applied heat unless countermeasures are implemented [151]. Another limitation of HT is that it can induce coagulation, particularly with perfusion or whole-body HT techniques, which may lead to hypoxia and contribute to tumor radioresistance [151].

**Figure 3:**
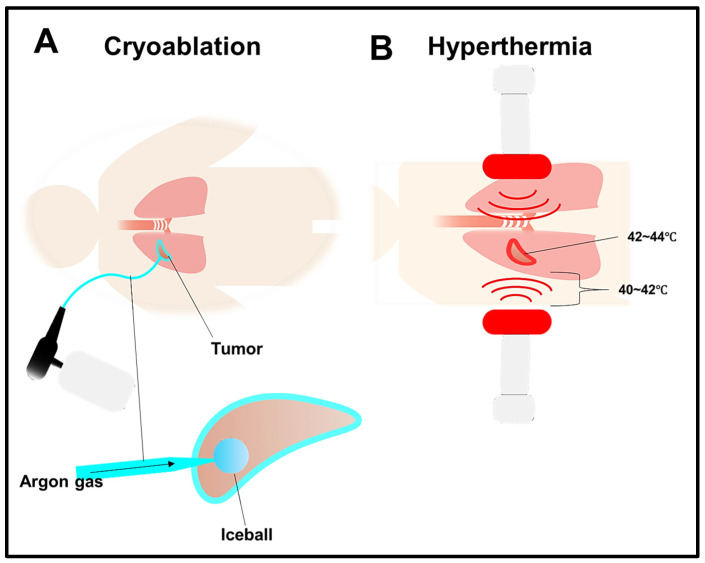
Simplified illustration showing how cryoablation and hyperthermia are carried out. (**A**) Cryoablation: During cryoablation, the cryoprobe forms ice balls and delivers an extremely cold freezing agent to the tumor site. A CT scan is employed to visualize the tumor and guide the precise placement of the ice ball around the targeted tumor. After treatment, a warmed cryoprobe is safely extracted from the patient; (**B**) hyperthermia involves heating the body tissue up to 44 °C to damage and eliminate cancer cells while minimizing harm to normal tissue. Small probes equipped with thermometers are inserted around the tumor to monitor and regulate the temperature closely. CT scans and other imaging techniques ensure proper probe placement.

HT combined with chemotherapy [149] or radiotherapy [152] has the potential to enhance the outcomes of lung cancer. HT serves as a supplementary or adjunctive therapy when used in conjunction with radiation and chemotherapy, particularly in the case of inoperable lung cancer [125]. For example, the combination of HT with radiation suppressed lung cancer progression in A549 cells and in vivo xenograft models [152]. Another study has shown that HT combined with glutathione administration facilitates the release of doxorubicin from a mesoporous silica nanocontainer drug carrier in lung cancer cells, ultimately leading to cell death [153]. Furthermore, the combination of cisplatin, cyclophosphamide, and HT in mice with metastatic Lewis lung carcinoma demonstrated a thermal potentiation of the growth delay induced by the combined drugs [154]. The prominent challenge associated with radiotherapy is the deposition of toxins into healthy tissues [151]. HT can minimize this effect by acting as a radiosensitizer and thus allowing a lower dose of radiotherapy [155]. In another clinical study, re-irradiation combined with HT decreased the level of toxicity in recurrent NSCLC patients and increased their long-term survival, particularly in cases without distant metastasis and those with larger recurrent tumors [156] (Figure 3).

## 8. Nanoparticles as a Tool for Targeted Therapy

Nanomedicine represents an emerging treatment approach focused on the enhancement of drug delivery and the optimization of therapeutic outcomes, with the aim of minimizing the harm to healthy tissues [157]. Biocompatibility and biodegradability bolster the effectiveness of nanomaterial-based drug delivery systems [158]. Concurrently, their therapeutic efficacy is enhanced by factors such as drug solubility, stability, and bioavailability [159]. Nanoparticles are also utilized to enhance cancer immunotherapy by boosting the immune response to cancer cells [160,161]. Some nanomedicine materials utilized in lung cancer treatment include hafnium oxide nanoparticles, magnetic nanoparticles, lipid nanoparticles, and polymer nanoparticles [162].

### 8.1. Hafnium Oxide Nanoparticles (HfO_2_ NPs) 

Hafnium oxide nanoparticles (HfO_2_ NPs) are utilized as both radiosensitizers and X-ray contrast agents due to their chemical inertness, high dielectric constant, elevated melting point, density, refractive index, and transparency to visible light, combined with minimal reactivity in biological systems [163]. HfO_2_ NPs are applicable in X-ray-induced photodynamic therapy (X-PDT) because they generate high-energy electrons and free radicals upon absorbing high-energy X-ray radiation [164]. NBTXR3, a type of HfO_2_ NP, was reported to help in treating metastatic lung cancer patients, irrespective of their sensitivity or resistance to immunotherapy [165]. Additionally, it has been shown to boost immune responses in a murine model of metastatic lung cancer resistant to anti-PD1 therapy [166]. The combination of immune therapy and a radiotherapeutic approach using NBTXR3 was shown to elicit the infiltration and activation of cytotoxic immune cells, leading to robust immunity in a dual-tumor model of lung cancer in mice [167]. Several clinical trials have been initiated, for example, an ongoing phase I/II clinical trial that involves the utilization of HfO_2_ NPs in combination with SABR and PD-1 inhibitors to treat metastatic NSCLC patients. The initial results indicate enhanced efficacy, but the trial is currently in its expansion phase [168].

### 8.2. Magnetic Nanoparticles (MNPs) 

Magnetic nanoparticles (MNPs) are made from materials with intrinsic magnetic properties, such as iron oxides, cobalt, and nickel. These MNPs can be employed in targeted drug delivery systems for lung cancer, offering significant drug-loading capabilities and effective tumor penetration [169]. Previous reports have shown that loading MNPs with cisplatin reduced the concentration of lung-resistance-related proteins, thereby enhancing cisplatin’s cytotoxicity in a cisplatin-resistant A549 cancer cell xenograft model [170]. Superparamagnetic iron oxide nanoparticles (SPIONs) could act as T2 contrast agents. When coated with oleic acid and carboxymethyl dextran and then conjugated with an anti-CD44v6 monoclonal antibody, they exhibit specific detection capabilities for metastatic lung cancer cells [171]. Moreover, in A549 human cancer cells, Fe_3_O_4_ MNPs coated with polyelectrolyte layers and loaded with doxorubicin hydrochloride chemotherapeutic drug exhibited cytotoxic effects [172]. MNPs can also be employed in magnetic hyperthermia by exposing them to an external magnetic field, which induces thermal effects to eliminate lung cancer cells without causing cytotoxic effects [173]. In conclusion, MNPs are applied in MRI contrasting, drug delivery, and hyperthermia therapy [159].

### 8.3. Lipid Nanoparticles (LNPs)

Lipid nanoparticles (LNPs) are composed of biocompatible lipids that encapsulate therapeutic compounds with diverse physicochemical properties that facilitate their absorption into cells and tissues. Subsequently, they optimize drug delivery to specific target areas in lung cancer, concurrently minimizing exposure to healthy tissues, thereby increasing the treatment efficacy and decreasing side effects [174]. One example of LNPs is liposomes characterized by an aqueous center enveloped by a lipid bilayer [175]. The liposomes are utilized to encapsulate hydrophilic drugs like cisplatin, resulting in enhanced drug delivery and increased anticancer effectiveness compared to free cisplatin in preclinical trials [176]. LNPs are employed for combining various drugs to combat drug resistance. For instance, transferrin-functionalized protein–lipid hybrid nanoparticles (PLHNs) containing both cisplatin and docetaxel effectively inhibited lung tumor growth in BALB/C mice with lung cancer [177]. Furthermore, a promising strategy for overcoming EGFR resistance entails the use of a pulmonary microsphere system. One study has shown that administering afatinib and paclitaxel to NSCLC cells resistant to EGFR TKIs using a pulmonary microsphere system led to effective treatment of drug-resistant lung cancer [178]. Moreover, targeted delivery facilitated by LNPs can serve as a tool for immunotherapy. For example, combining an anti-PD-1 antibody with LNPs loaded with a STING agonist enhances the NK cell activity in lung metastatic tumors compared to using anti-PD-1 alone [179].

### 8.4. Polymeric Nanoparticles (PNPs)

Polymeric nanoparticles (PNPs) comprise synthetic and natural polymers for targeted drug delivery in lung cancer [180]. PNPs have shown enhanced drug release, biocompatibility, and increased anticancer effects due to their composition of polylactic acid (PLA), polyethylene glycol (PEG), poly(lactic-co-glycolic acid) (PLGA), chitosan-loaded lomustine, and gelatin conjugated with biotinylated epidermal growth factor (EGF) [181]. In lung cancer, nitroimidazole- and hyaluronic-acid-based PNPs and lipid nanoparticles (PNP-LNP hybrids) are utilized for the targeted delivery of cisplatin to lung cancer cells and xenografts, resulting in a potent anti-tumor response while minimizing toxicity [182]. Additionally, another study illustrated that sorafenib-loaded polynanoparticles (PNPs) with cationic modifications enhanced the therapeutic effectiveness of sorafenib in NSCLC when compared to the administration of the drug alone [183]. Folate receptor-targeting multifunctional dual drug-loaded nanoparticles (MDNPs) consists of a poly(N-isopropylacrylamide)-carboxymethyl chitosan shell and a PLGA core. These NPs are employed for the efficient delivery of chemo–radiotherapy for lung cancer. For instance, MDNPs applied to the controlled release of a NU7441 radiosensitizer and the chemotherapeutic drug gemcitabine showed minimal toxicity and increased efficacy in lung cancer cells [184]. In combination therapy, PNPs serve as radiosensitizers by combining doxorubicin-loaded polyaspartamide PNPs with 5-aminolevulinic acid to target lung cancer cells, resulting in selective radiosensitization and enhanced efficacy [185]. Therefore, PNPs offer a promising approach to treating lung cancer by improving treatment efficacy with minimal toxicity. 

## 9. Conclusions

**Figure 4:**
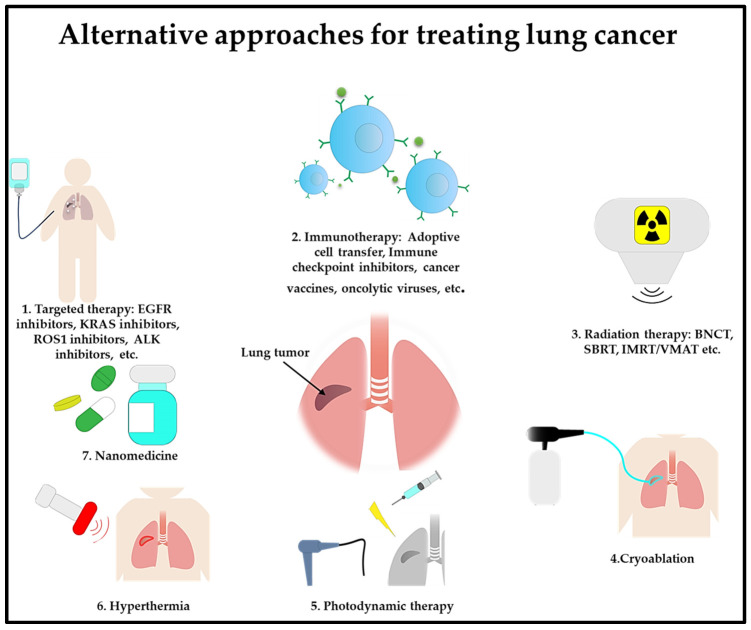
Emerging lung cancer treatments are targeted therapy, immunotherapy, radiation therapy, cryoablation, photodynamic therapy, hyperthermia, and nanomedicine.

In summary, the field of lung cancer treatment is experiencing a profound transformation, marked by the introduction of groundbreaking therapies (Figure 4). Advancements in personalized medicine, targeted therapies, and immunotherapy provide new hope for patients with this challenging disease. A combination approach to using these cutting-edge therapies could enhance lung cancer treatment by boosting the treatment effectiveness while minimizing toxicity effects. As scientific research delves deeper into the intricacies of the disease, continuous progress in emerging therapies for lung cancer has the potential to redefine the standard of care.

## Figures and Tables

**Table 2:** Representative antibodies used as immune checkpoint inhibitors in lung cancer.

Representative Antibody	Target	Approved Indication	Refs.
Nivolumab	PD-1	Advanced NSCLC	[86]
Pembrolizumab	PD-1 and PD-2	Advanced NSCLC	[87]
Cemiplimab	PD-1	Advanced NSCLC	[88]
Atezolizumab	PD-1 and PD-L1	NSCLC	[89,90]
Durvalumab	PD-L1	Advanced NSCLC	[91]
Ipilimumab	CTLA-4	Advanced NSCLC	[86]
Tremelimumab	CTLA-4	Metastatic NSCLC	[92]
